# Morphological characteristics and phylogenetic analyses revealed four new wood-inhabiting species of *Botryobasidium* (*Cantharellales*, *Basidiomycota*) from East China

**DOI:** 10.3897/mycokeys.139.206012

**Published:** 2026-09-02

**Authors:** Wen Jing, Wan-Ying Li, Viktor Papp, Qian-Xin Guan, Zi-Wei Zheng, Yu-Jin Cui, Fang Wu

**Affiliations:** 1 School of Ecology and Nature Conservation, Beijing Forestry University, Beijing 100083, China School of Ecology and Nature Conservation, Beijing Forestry University Beijing China; 2 Department of Botany, Hungarian University of Agriculture and Life Sciences, H-1118 Budapest, Hungary Department of Botany, Hungarian University of Agriculture and Life Sciences Budapest Hungary; 3 Department of Plant Anatomy, Institute of Biology, Eötvös Loránd University, H-1117 Budapest, Hungary Department of Plant Anatomy, Institute of Biology, Eötvös Loránd University Budapest Hungary

**Keywords:** *
Botryobasidiaceae
*, new species, phylogeny, taxonomy

## Abstract

Four new species of *Botryobasidium*, *B. fujianensis*, *B. fusisporellum*, *B. subconiferarum* and *B. macrosporum*, are described from east China based on morphological characteristics and phylogenetic analyses using the combined dataset of ITS+nLSU sequences. *Botryobasidium fujianensis* is characterised by a hypochnoid, floccose to cottony, white to greyish-white hymenial surface, a monomitic hyphal system with simple septa, the presence of tubular cystidioid, barrel-shaped basidia with four or six sterigmata and pip-shaped basidiospores measuring 6.0–7.9 × 2.3–3.4 μm; *B. fusisporellum* is characterised by a hypochnoid, floccose to cottony, white to greyish-white hymenial surface, a monomitic hyphal system with simple septa, the presence of tubular cystidioid, long barrel-shaped basidia with two or four sterigmata and fusiform basidiospores measuring 5.3–6.5 × 2.3–3.0 μm; *B. subconiferarum* is characterised by a hypochnoid, floccose, white to greyish-white hymenial surface, a monomitic hyphal system with simple septa, the presence of tubular cystidioid, slightly barrel-shaped basidia with four sterigmata, subcylindrical conidiophores and pip-shaped basidiospores measuring 6.2–8.6 × 2.3–3.7 μm; *B. macrosporum* is characterised by a hypochnoid, floccose, white to cream hymenial surface, a monomitic hyphal system with simple septa, the presence of tubular cystidioid, slightly barrel-shaped basidia with four to six sterigmata and fusiform basidiospores measuring 9.4–11.0 × 4.8–5.9 μm. The discovery of these four new species in east China expands the known global diversity of *Botryobasidium* and provides additional morphological and molecular data for the genus.

## Introduction

Wood-inhabiting fungi degrade cellulose and lignin, contributing to wood decomposition. They facilitate the nutrient cycling, carbon turnover and energy flow in forests, with some species also possessing edible and medicinal value (Wu et al. 2022a; Dong et al. 2023, 2024; Ponce et al. 2023; Zhao et al. 2024; Dai et al. 2025; Yang et al. 2025; Zhang et al. 2025). Within this ecological group, species of *Botryobasidium* Donk (*Botryobasidiaceae*, *Cantharellales*) are predominantly white-rot fungi, which result in white-rotted decay of the decomposed wood (Moncalvo et al. 2006; Xiong et al. 2009; Li et al. 2025). They commonly occur on rotten wood and fallen branches of angiosperms and gymnosperms, such as *Dendrocalamus* spp., *Leucaena* spp., *Hippophae* spp., *Quercus* spp., *Abies* spp., *Pinus* spp., *Picea* spp. etc. (Langer et al. 2000a, 2000b; Hyde et al. 2019; Dong et al. 2024; Zhou et al. 2024a, 2024b; Li et al. 2025). Some species have also been reported from soil or from mature basidiomata of *Irpex lacteus* (Fr.) Fr. (Anonymous 1969; Boidin and Gilles 1982).

*Botryobasidium* is typified by *B. subcoronatum* (Höhn. & Litsch.) Donk and is characterised by annual, resupinate basidiomata with smooth, pellicular, hypochnoid or arachnoid hymenophores; a monomitic hyphal system with simple septa or clamp connections and branching mostly at right angles; basidia with 2–8 sterigmata; and smooth or ornamented basidiospores (Donk 1931, 1964; Langer et al. 2000a, 2000b; Moncalvo et al. 2006; Xiong et al. 2009; Li et al. 2025). The genus was initially proposed by Donk (1931), based on morphological studies, in which he observed that the basidia bear four sterigmata and the basidiospores are densely ornamented with rodlets. Subsequently, Langer (1994) conducted a comprehensive worldwide morphological revision of *Botryobasidium*. On the basis of this work and later studies, 49 species were accepted in the genus (Langer et al. 2000a, 2000b). Amongst them, many *Botryobasidium* species exhibited anamorphic stages (Bernicchia and Gorjón 2010). Phylogenetic studies have demonstrated that *Botryobasidium* forms a well-supported monophyletic group (Hibbett et al. 1997; Hyde et al. 2019). *Neoacladium* P.N. Singh & S.K. Singh, known only from its asexual morph, was proposed as a sister to *Botryobasidium*, based on the morphological characters and phylogenetic analysis of ITS and nLSU sequence data (Hyde et al. 2019). Based mainly on asexual morphology, Stalpers et al. (2021) assigned 22 species originally described in other genera, including *Acladium* Link, *Haplotrichum* Link and *Neoacladium*, to *Botryobasidium*. However, most of these taxonomic placements have not yet been confirmed by molecular data. Recently, some species have been newly described in *Botryobasidium*, based on both sexual morphology and phylogenetic analyses of ITS and nLSU sequence data, including *B. acanthosporum* L.Jiang Zhou & H.S. Yuan, *B. daweishanense* J.L. Zhang et al., *B. leptocystidiatum* L.Jiang Zhou & H.S. Yuan, *B. latihyphum* Xin Li et al. and *B. zhejiangensis* Xin Li et al. (Zhou et al. 2024a, 2024b; Li et al. 2025; Zhang et al. 2025). These studies consistently recovered *Botryobasidium* as a strongly supported monophyletic group.

According to Index Fungorum (www.indexfungorum.org; accessed on 11 August 2026) and the MycoBank database (http://www.MycoBank.org; accessed on 11 August 2026), 120 names of *Botryobasidium* have been recorded worldwide. However, some of these names are invalidly published or transferred to other genera. Amongst them, 34 species of *Botryobasidium* have been confirmed by molecular sequence data and 22 species have been reported in China (Langer et al. 2000a, 2000b; Parmasto et al. 2004; Bernicchia et al. 2010; Stalpers et al. 2021; Dong et al. 2024, 2025; Zhou et al. 2024a, 2024b; Li et al. 2025; Zhang et al. 2025;Yuan et al. 2026).

During field surveys of wood-inhabiting fungi in east China, six specimens morphologically corresponding to *Botryobasidium* were collected. Phylogenetic analysis, based on ITS and nLSU sequences, was conducted to assess their placement within the genus. The six studied specimens formed four distinct lineages within the *Botryobasidium* clade and were distinguishable from their phylogenetically related and morphologically similar species. Based on the combined morphological and phylogenetic evidence, they are described here as four new species of *Botryobasidium*.

## Materials and methods

### Morphological studies

The examined specimens were stored at the Fungarium of Beijing Forestry University (**BJFC**). The descriptions of macro-morphology were based on field records during specimen collection and laboratory observations. Microscopic measurements and drawings were made from slide preparations of dried tissues stained with Cotton Blue and Melzer’s reagent.

The following abbreviations were used: IKI = Melzer’s reagent; IKI– = both inamyloid and undextrinoid in Melzer’s reagent; KOH = 5% potassium hydroxide; CB = cotton blue; CB– = acyanophilous; CB+ = cyanophilous; L = mean basidiospore length (arithmetic average of all measured basidiospores); W = mean basidiospore width (arithmetic average of all measured basidiospores); Q = L/W ratio for each specimen studied; and n (a/b) = number of basidiospores (a) measured from a given number (b) of specimens. Special colour terms followed Anonymous (1969) and Petersen (1996).

### DNA extraction and sequencing

The total genomic DNA was extracted from dried samples using a CTAB rapid plant genome extraction kit-DNA (Aidlab Biotechnologies, Co., Ltd., Beijing, China) according to the manufacturer’s instructions, with some modifications (Wu et al. 2022b). The ITS region was amplified using primers ITS4 and ITS5, while the nLSU region was amplified using primers LR0R and LR7 (White et al. 1990). The PCR procedure for ITS was as follows: initial denaturation at 95 °C for 3 min, followed by 35 cycles at 94 °C for 40 s, 54 °C for 45 s and 72 °C for 1 min, with a final extension at 72 °C for 10 min. The PCR procedure for nLSU was as follows: initial denaturation at 94 °C for 1 min, followed by 35 cycles of denaturation at 94 °C for 30 s, annealing at 50 °C for 1 min and extension at 72 °C for 1.5 min and a final extension at 72 °C for 10 min. The PCR products were purified and sequenced at the Beijing Genomics Institute, China, using the same primers as those in the PCR reactions.

### Phylogenetic analysis

Taxon names, voucher information and GenBank accession numbers of ITS and nLSU sequences used in this study are shown in the phylogenetic tree. In the phylogenetic analysis of the combined dataset of ITS+nLSU sequences, *Lyomyces allantosporus* Riebesehl, Yurchenko & Langer and *L. pruni* (Lasch) Riebesehl and Langer were chosen as outgroups following previous studies (Zhou et al. 2024b; Li et al. 2025). Reference sequences retrieved from GenBank, chosen to include published species with DNA data and sequences from earlier research and the newly-generated sequences were partitioned into ITS1, 5.8S, ITS2 and nLSU regions. Each dataset was aligned separately using MAFFT v.7.526 with the G-INS-i iterative refinement algorithm (Katoh et al. 2019; https://mafft.cbrc.jp/alignment/server/) and manually adjusted in BioEdit v.7.0.5.3 (Hall 1999). The aligned datasets were subsequently concatenated using PhyloSuite v.1.2.2 (Zhang et al. 2020). The phylogenetic tree of *Botryobasidium* was constructed using the concatenated ITS1+5.8S+ITS2+nLSU dataset and phylogenetic analysis was conducted through Maximum Likelihood (ML) and Bayesian Inference (BI). The final alignment and resulting topologies were deposited in TreeBASE (http://www.treebase.org) under accession 32752.

ML analysis was performed with RAxML v.7.2.8 under the GTR+I+G model of site substitution, including estimation of gamma-distributed rate heterogeneity and a proportion of invariant sites (Stamatakis 2006). Branch support was assessed employing the bootstrap method with 1,000 replicates (Hillis and Bull 1993).

BI analysis was performed using MrBayes 3.2.7 with the best-fit partitioning scheme and substitution model determined by ModelFinder v.2.2.0 (Ronquist et al. 2012; Kalyaanamoorthy et al. 2017). Four Markov chains were run for two independent runs from random starting trees for one million generations, until the split deviation frequency value reached < 0.01 and trees were sampled every 1,000 generations. The first 25% of the sampled trees were discarded as burn-in and the remaining trees were used to reconstruct a majority-rule consensus tree and to calculate Bayesian posterior probabilities (BPP) for the resulting clades. Phylogenetic trees were visualised in FigTree v.1.4.4 (Rambaut 2018). Branches receiving ML bootstrap support (BS) and BPP values ≥ 75% and ≥ 0.90, respectively, were shown at the nodes.

In addition, the phylogenetic tree of *Botryobasidium* was constructed using a concatenated dataset of ITS1+5.8S+ITS2 to further validate our phylogenetic analysis results. The resulting phylogenetic tree is provided as Suppl. material 1.

## Results

### Phylogenetic analyses

**Figure 1.**
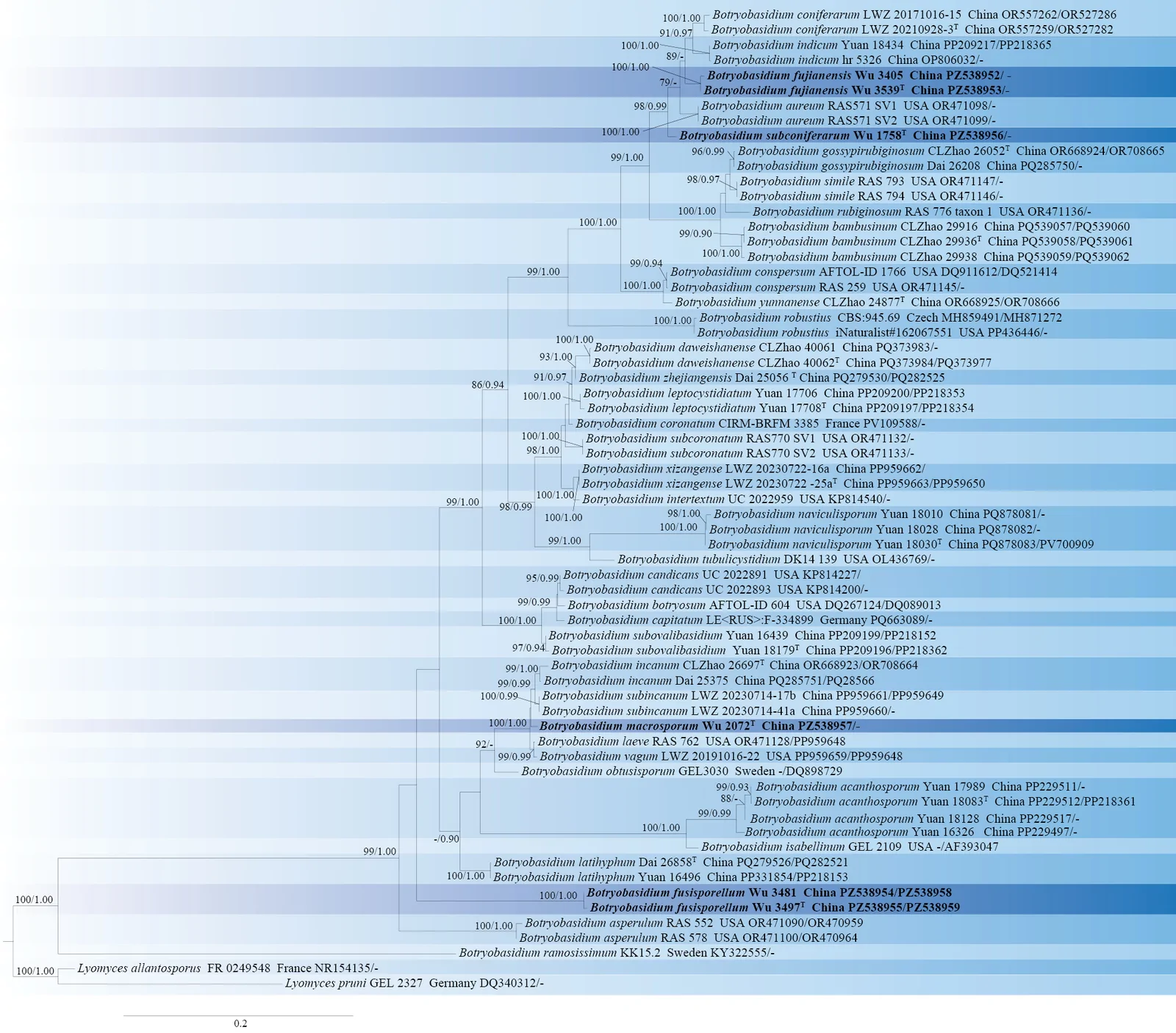
Maximum Likelihood (ML) phylogenetic tree illustrating the phylogeny of *Botryobasidium*, based on the combined ITS1+5.8S+ITS2+nLSU dataset. Branches are labelled with ML bootstrap values (BS) greater than 75% and Bayesian posterior probabilities greater than 0.90. The new species are shown in bold, ^T^ indicates type material, holotype.

In the phylogenetic analysis of *Botryobasidium*, the combined ITS1+5.8S+ITS2+nLSU dataset comprised sequences from 65 fungal specimens representing 39 taxa. The final alignment contained a total of 2,101 nucleotide positions, including 283 bases of ITS1, 158 bases of 5.8S, 327 bases of ITS2 and 1,333 bases of nLSU. The nLSU region was relatively conserved, whereas the ITS region was more variable. The best-fit substitution models proposed by ModelFinder for the Bayesian analysis were GTR+F+G4 for ITS1, K2P+G4 for 5.8S, GTR+F+G4 for ITS2 and GTR+F+I+G4 for nLSU. The BI analysis resulted in an average standard deviation of split frequencies of 0.005707. Due to the similarity in topology between ML and BI trees, only the topology of the ML analysis is presented, with the statistical values of the ML (≥ 75%) and Bayesian posterior probabilities (BPP ≥ 0.90) shown (Fig. 1).

In the phylogenetic tree inferred from the combined ITS1+5.8S+ITS2+nLSU dataset (Fig. 1), our newly-collected specimens formed four well-supported distinct lineages, representing four new species, viz. *Botryobasidium fujianensis*, *B. fusisporellum*, *B. subconiferarum* and *B. macrosporum*. The two specimens of *B. fujianensis* grouped with *B. aureum* Parmasto, *B. indicum* (P.N. Singh & S.K. Singh) R. Kirschner and G. Langer, *B. coniferarum* S.L. Liu & L.W. Zhou and *B. subconiferarum* with strong support (98/0.99). The two specimens of *B. fusisporellum* formed an independent lineage within *Botryobasidium* with high support (100/1.00), which is not related to other *Botryobasidium* species. The single specimen of *B. subconiferarum* formed a distinct lineage within *Botryobasidium*. The single specimen of *B. macrosporum* formed a distinct lineage which is closely related to *B. laeve* (J. Erikss.) Parmasto, *B. vagum* (Berk. & M.A. Curtis) D.P. Rogers, *B. incanum* Qian Zhou & C.L. Zhao and *B. subincanum* S.L. Liu & L.W. Zhou with high support (100/1.00).

In addition, the phylogenetic tree inferred from the ITS1+5.8S+ITS2 dataset showed a topology broadly congruent with that obtained from the combined ITS1+5.8S+ITS2+nLSU dataset. This analysis included 63 fungal specimens representing 37 taxa and is presented in Suppl. material 1.

Pairwise ITS sequence divergence between *Botryobasidium fujianensis* and its closest relatives was 5.55% from *B. aureum*, 6.56% from *B. coniferarum*, 7.03% from *B. indicum* and 5.13% from *B. subconiferarum*. *Botryobasidium subconiferarum* differed by 4.46% from *B. aureum*, 6.70% from *B. coniferarum*, 5.13% from *B. fujianensis* and 5.36% from *B. indicum*. *Botryobasidium macrosporum* differed by 3.50% from *B. incanum*, 3.15% from *B. laeve*, 2.98% from *B. subincanum* and 3.85% from *B. vagum*.

### Taxonomy

#### Botryobasidium fujianensis

Taxon classificationFungiCantharellalesBotryobasidiaceae

F. Wu, W. Jing, W.Y. Li & Y.C. Dai sp. nov.

780A4A1C-01E1-5046-920A-E9E305ABC933

864124

Figs 2a, 3

##### Diagnosis.

*Botryobasidium fujianensis* is distinguished by its annual, resupinate, adnate, hypochnoid basidiomata; monomitic hyphal structure, simple septate generative hyphae; tubular cystidioid, barrel-shaped basidia with four or six sterigmata; pip-shaped basidiospores (6.0–7.9 × 2.3–3.4 μm).

**Figure 2.**
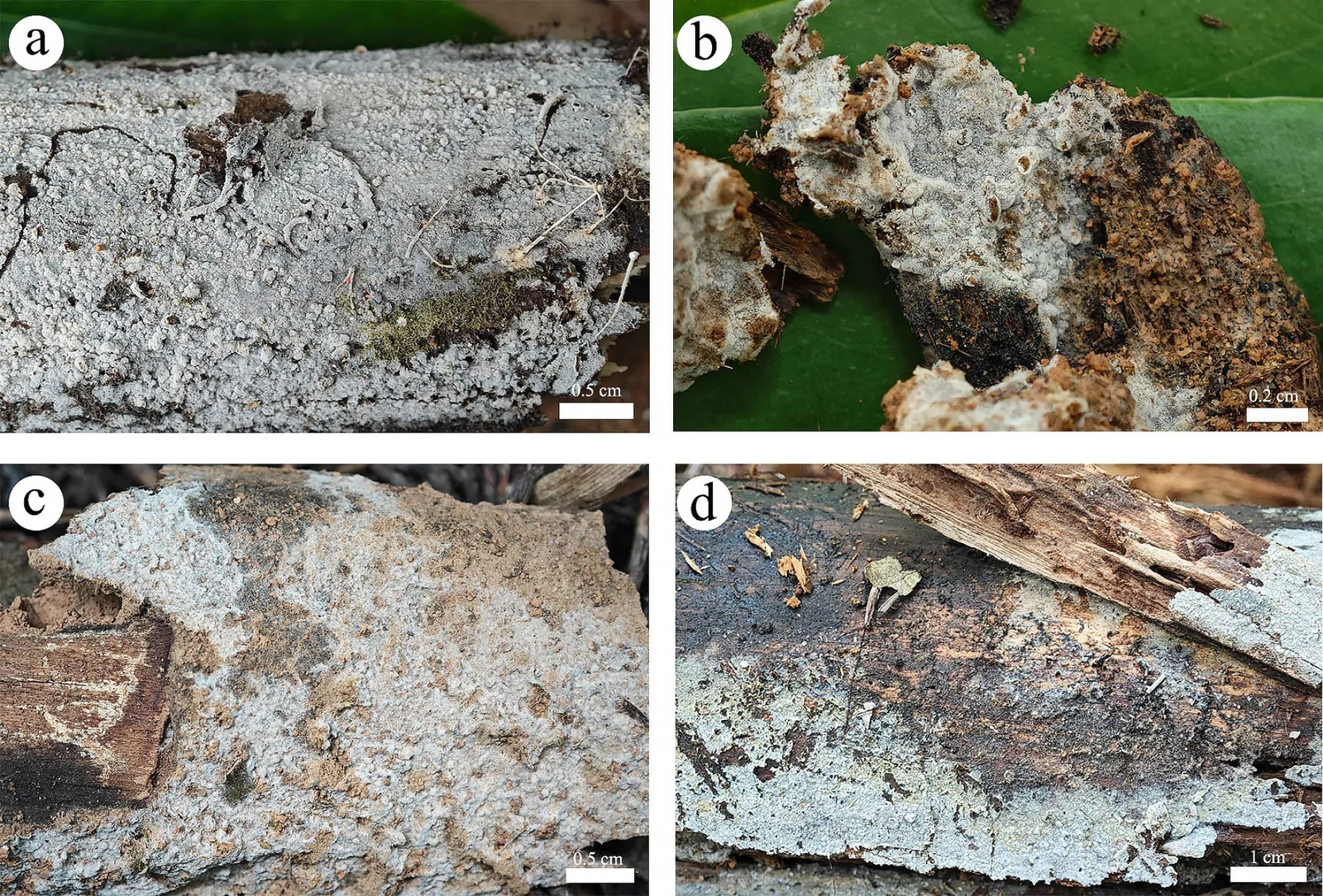
Basidiomata of four new species of *Botryobasidium*. **a**. *B. fujianensis* (holotype, Wu 3539); **b**. *B. fusisporellum* (holotype, Wu 3497); **c**. *B. subconiferarum* (holotype, Wu 1758); **d**. *B. macrosporum* (holotype, Wu 2072).

**Holotype**. China • Fujian Province, Nanping, Wuyishan National Nature Reserve, 27°43'51"N, 117°42'50"E, 586 m a.s.l., 20 July 2025, on rotten wood of *Cunninghamia*, F. Wu leg., Wu 3539 (BJFC 063655, holotype).

##### Etymology.

*Fujianensis* (Lat.): Referring to the species being found in Fujian Province, east China.

##### Description.

***Basidiomata***. Annual, resupinate, adnate, hypochnoid, up to 10.5 cm long, 5 cm wide, 0.2 mm thick at centre, without odour and taste when fresh and dry; hymenial surface floccose to cottony and occasionally granulose, white to greyish-white when fresh, uncracked, cream to straw-yellow when dry; sterile margin indistinct, thinning out, concolorous with hymenial surface.

**Figure 3.**
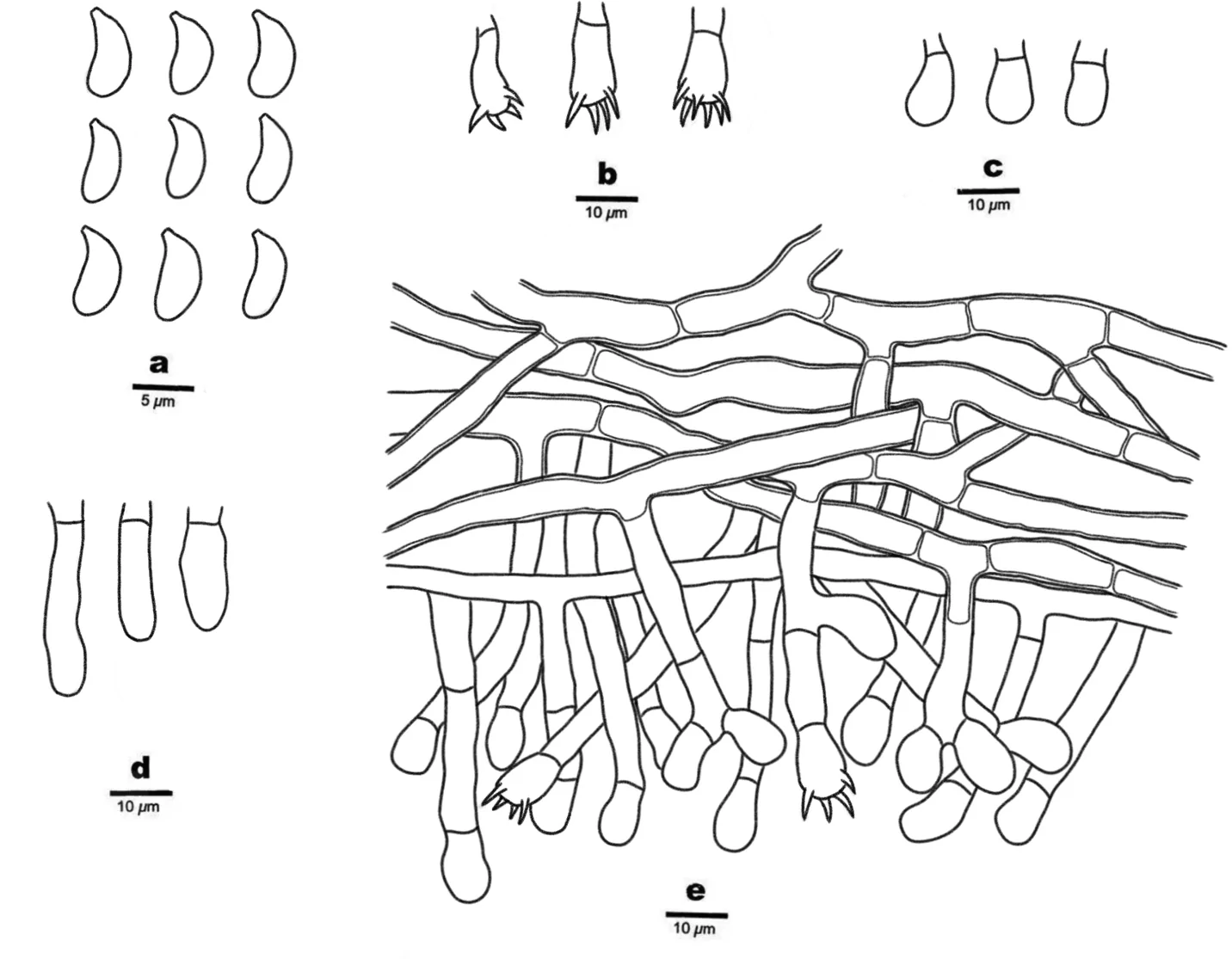
Microscopic structures of *Botryobasidium fujianensis* (holotype, Wu 3539). **a**. Basidiospores; **b**. Basidia; **c**. Basidioles; **d**. Cystidioid; **e**. A section of basidiomata. Scale bars: 5 μm (**a**); 10 μm (**b–e**).

***Hyphal structure***. Monomitic, generative hyphae simple septate, CB+, IKI–, tissues unchanged in KOH; subhymenial hyphae hyaline, thin-walled, smooth, frequently branched at right angles, loosely interwoven, 4–7 µm in diam.; subicular hyphae hyaline, slightly thick-walled, smooth, frequently branched at right angles, 6–10 µm in diam.

***Hymenium***. Cystidioid tubular, hyaline, thin-walled, smooth, with a basal simple septum, apically obtuse, 24–35 × 5–10 μm; basidia barrel-shaped, hyaline, thin-walled, smooth, with four or six sterigmata and a basal simple septum, 12–20 × 6–9 μm; basidioles in shape similar to basidia, but slightly smaller.

***Basidiospores***. Pip-shaped, hyaline, thin-walled, smooth, CB+, IKI–, (5.6–)6.0–7.9(–8.4) × (2.1–)2.3–3.4(–3.5) μm, L = 6.89 μm, W = 2.88 μm, Q = 2.39–2.41 (n = 60/2).

##### Ecology and distribution.

Growing on coniferous trees in subtropical regions. So far, known from Fujian Province, China.

##### Additional specimen examined

**(paratype)**. China • Fujian Province, Sanming, Yongan County, Tianbaoyan National Nature Reserve, 25°57'15"N, 117°32'9"E, 759 m a.s.l., 17 July 2025, on fallen branch of *Pinus*, F. Wu leg., Wu 3405 (BJFC 063521).

#### Botryobasidium fusisporellum

Taxon classificationFungiCantharellalesBotryobasidiaceae

F. Wu, W. Jing, W.Y. Li & Y.C. Dai sp. nov.

B4BB4936-BAFA-52C9-A35B-02C51C3D88DF

864125

Figs 2b, 4

##### Diagnosis.

*Botryobasidium fusisporellum* is distinguished by annual, resupinate, adnate, hypochnoid basidiomata; monomitic hyphal structure, simple septate generative hyphae; tubular cystidioid, long barrel-shaped basidia with two or four sterigmata; fusiform basidiospores (5.3–6.5 × 2.3–3.0 μm).

**Holotype**. China • Fujian Province, Nanping, Jianou County, Wanmulin Nature Reserve, 27°3'3"N, 118°8'49"E, 353 m a.s.l., 19 July 2025, on rotten wood, F. Wu leg., Wu 3497 (BJFC 063613, holotype).

##### Etymology.

*Fusisporellum* (Lat.): Referring to the species having fusiform basidiospores.

##### Description.

***Basidiomata***. Annual, resupinate, adnate, hypochnoid, up to 6 cm long, 3 cm wide, 0.35 mm thick at centre, without odour and taste when fresh and dry; hymenial surface floccose to cottony, white to greyish-white when fresh, uncracked, greyish-white to cream when dry; sterile margin indistinct, thinning out, concolorous with hymenial surface.

**Figure 4.**
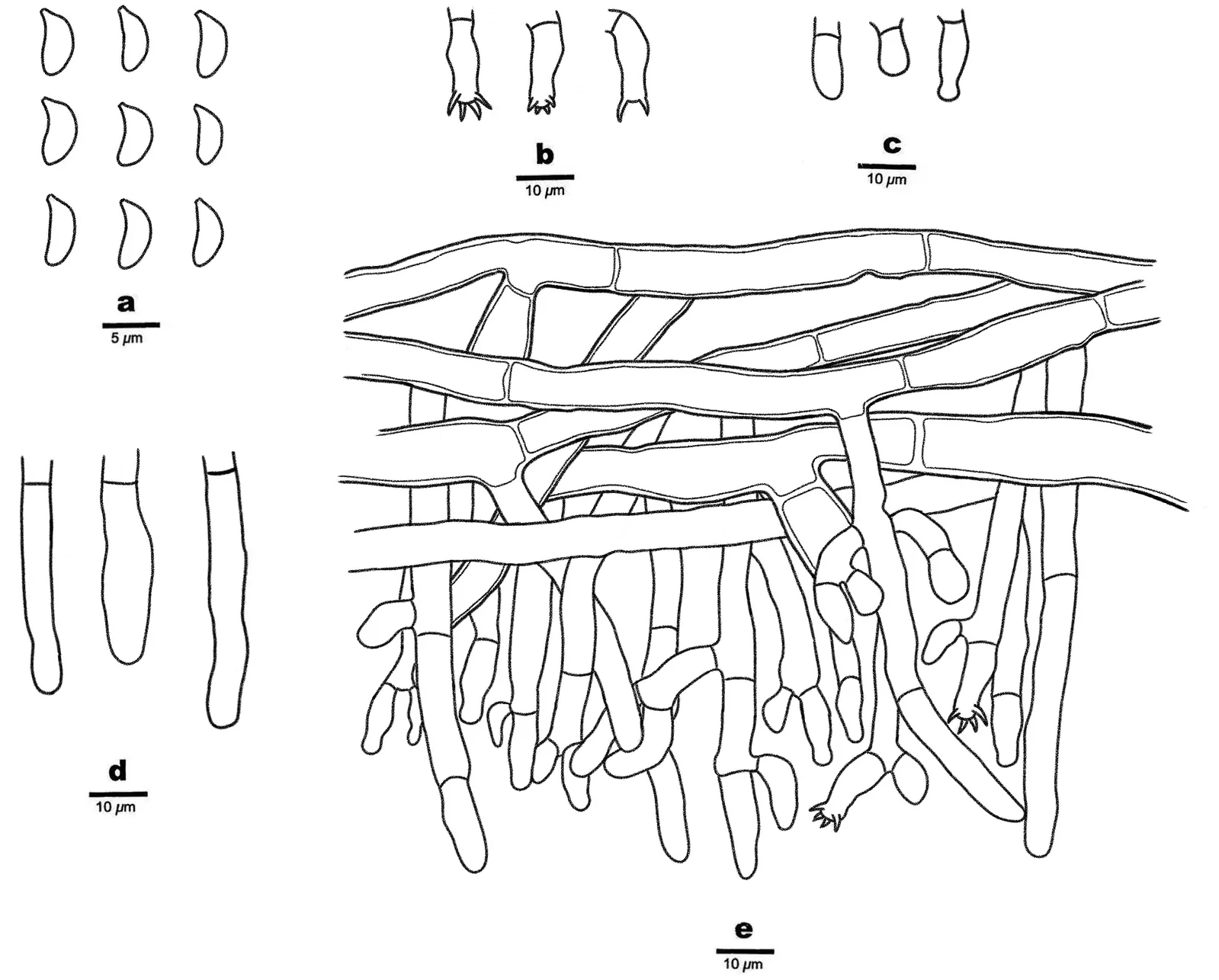
Microscopic structures of *Botryobasidium fusisporellum* (holotype, Wu 3497). **a**. Basidiospores; **b**. Basidia; **c**. Basidioles; **d**. Cystidioid; **e**. A section of basidiomata. Scale bars: 5 μm (**a**); 10 μm (**b–e**).

***Hyphal structure***. Monomitic, generative hyphae simple septate, CB+, IKI–; tissues unchanged in KOH; subhymenial hyphae hyaline, thin-walled, smooth, frequently branched at right angles, loosely interwoven, 5–9 µm in diam.; subicular hyphae hyaline, slightly thick-walled, smooth, frequently branched at right angles, 9–13 µm in diam.

***Hymenium***. Cystidioid tubular, hyaline, thin-walled, smooth, with a basal simple septum, apically obtuse, infrequent, 23–55 × 5–8 μm; Basidia long barrel-shaped, hyaline, thin-walled, with two or four sterigmata and a basal simple septum, 14–22 × 4–7 μm; basidioles in shape similar to basidia, but smaller.

***Basidiospores***. Fusiform, hyaline, thin-walled, smooth, CB+, IKI–, (5.2–)5.3–6.5(–6.8) × (2.2–)2.3–3.0(–3.2) μm, L = 5.99 μm, W = 2.61 μm, Q = 2.29–2.31 (n = 60/2).

##### Ecology and distribution.

Growing on rotten wood and soil over stone in subtropical region. So far, known from Fujian Province, China.

##### Additional specimen examined

**(paratype)**. China • Fujian Province, Nanping, Jianou County, Wanmulin Nature Reserve, 27°3'7"N, 118°8'54"E, 350 m a.s.l., 19 July 2025, on soil over stone, F. Wu leg., Wu 3481 (BJFC 063597).

#### Botryobasidium subconiferarum

Taxon classificationFungiCantharellalesBotryobasidiaceae

F. Wu, W. Jing, W.Y. Li & Y.C. Dai sp. nov.

FD627F53-2D67-5697-9F4B-8CC61BF2B306

864126

Figs 2c, 5

##### Diagnosis.

*Botryobasidium subconiferarum* is distinguished by annual, resupinate, adnate, hypochnoid basidiomata; monomitic hyphal structure, simple septate generative hyphae; tubular cystidioid, slightly barrel-shaped basidia with four sterigmata; subcylindrical conidiophores; pip-shaped basidiospores (6.2–8.6 × 2.3–3.7 μm).

**Holotype**. China • Jiangxi Province, Jiujiang, Lianxi District, Tianhuajing National Forest Park, 29°39'8"N, 116°1'10"E, 125 m a.s.l., 10 July 2024, on fallen angiosperm branch, F. Wu leg., Wu 1758 (BJFC 046067, holotype).

##### Etymology.

*Subconiferarum* (Lat.): Referring to the species having conidiophores and similar to *Botryobasidium coniferarum*.

##### Description.

***Basidiomata***. Annual, resupinate, adnate, hypochnoid, up to 7 cm long, 4 cm wide, 0.15 mm thick at centre, without odour and taste when fresh and dry; hymenial surface floccose, white to greyish-white when fresh, uncracked, straw-yellow to olivaceous buff when dry; sterile margin indistinct, thinning out, concolorous with hymenial surface.

**Figure 5.**
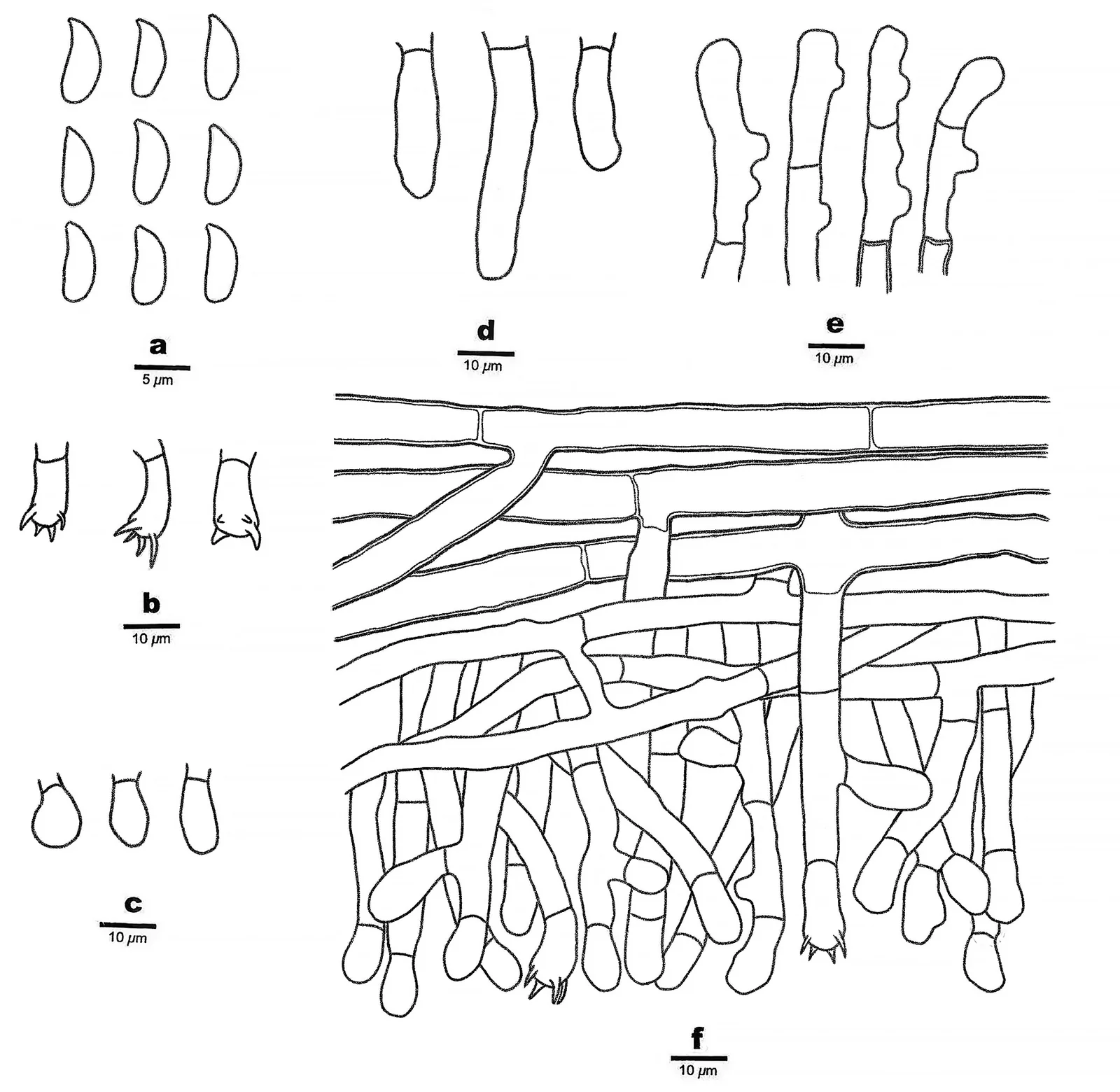
Microscopic structures of *Botryobasidium subconiferarum* (holotype, Wu 1758). **a**. Basidiospores; **b**. Basidia; **c**. Basidioles; **d**. Cystidioid; **e**. Conidiophores; **f**. A section of basidiomata. Scale bars: 5 μm (**a**); 10 μm (**b–f**).

***Hyphal structure***. Monomitic, generative hyphae simple septate, CB+, IKI–; tissues unchanged in KOH; subhymenial hyphae hyaline, thin-walled, smooth, frequently branched at right angles, loosely interwoven, 5–7 µm in diam.; subicular hyphae hyaline, slightly thick-walled, smooth, frequently branched at right angles, 7–9 µm in diam.

***Hymenium***. Cystidioid tubular, hyaline, thin-walled, smooth, with a basal simple septum, apically obtuse, infrequent, 18–36 × 6–8 μm; basidia slightly barrel-shaped, hyaline, thin-walled, with four sterigmata and a basal simple septum, 14–21 × 6–10 μm; basidioles in shape similar to basidia, but slightly smaller.

***Conidiophores***. Subcylindrical, hyaline, thin-walled, relatively uniform in diameter or gradually tapering distally, with a basal simple septum, up to 54 µm long, 5–10 µm in diameter; distal position straight, with sporogenous teeth borne mainly on the apical cells.

***Basidiospores***. Pip-shaped, hyaline, thin-walled, smooth, CB+, IKI–, (5.5–)6.2–8.6(–8.7) × (2.1–)2.3–3.7(–3.8) μm, L = 7.35 μm, W = 3.06 μm, Q = 2.42 (n = 50/1).

##### Ecology and distribution.

Growing on fallen angiosperm branch in subtropical region. So far, known from Jiangxi Province, China.

#### Botryobasidium macrosporum

Taxon classificationFungiCantharellalesBotryobasidiaceae

F. Wu, W. Jing, W.Y. Li & Y.C. Dai sp. nov.

F74BAEFD-C1BD-57A3-AB37-4481A720AD18

864127

Figs 2d, 6

##### Diagnosis.

*Botryobasidium macrosporum* is distinguished by annual, resupinate, adnate, hypochnoid basidiomata; monomitic hyphal structure, simple septate generative hyphae; tubular cystidioid, slightly barrel-shaped basidia with four to six sterigmata; fusiform basidiospores (9.4–11.0 × 4.8–5.9 μm).

**Holotype**. China • Anhui Province, Anqing, Yuexi County, Laibang Town, 30°54'32"N, 116°14'8"E, 724 m a.s.l., 4 July 2024, on angiosperm trunk, F. Wu leg., Wu 2072 (BJFC 046380, holotype).

##### Etymology.

*Macrosporum* (Lat.): Referring to the species having large basidiospores.

##### Description.

***Basidiomata***. Annual, resupinate, adnate, hypochnoid, up to 13 cm long, 4 cm wide, 0.6 mm thick at centre, without odour and taste when fresh and dry; hymenial surface floccose and occasionally granulose, white to cream when fresh, uncracked, cream to greyish-white when dry; sterile margin indistinct, thinning out, concolorous with hymenial surface.

**Figure 6.**
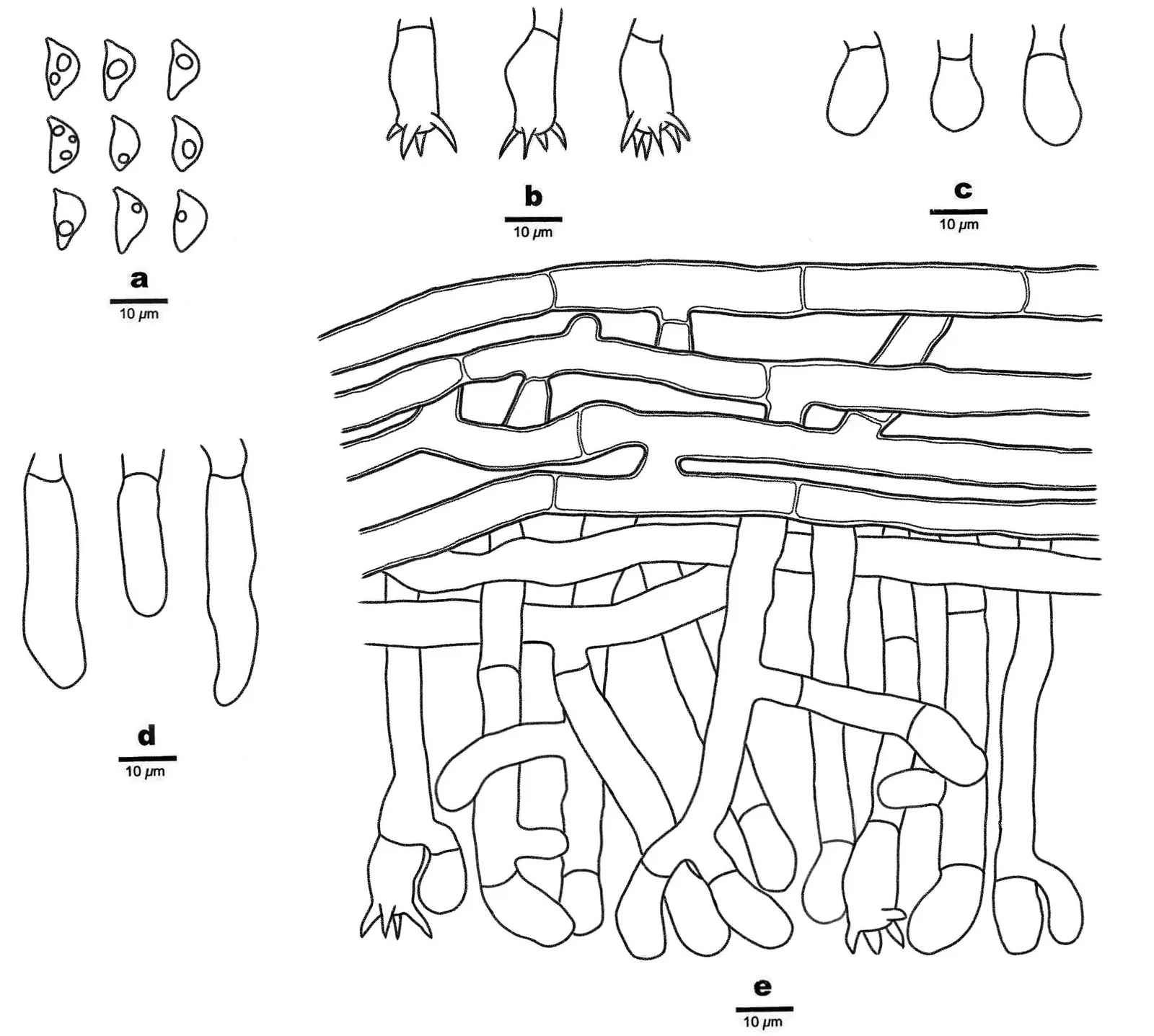
Microscopic structures of *Botryobasidium macrosporum* (holotype, Wu 2072). **a**. Basidiospores; **b**. Basidia; **c**. Basidioles; **d**. Cystidioid; **e**. A section of basidiomata. Scale bars: 10 μm.

***Hyphal structure***. Monomitic, generative hyphae simple septate, CB+, IKI–; tissues unchanged in KOH; subhymenial hyphae hyaline, thin-walled, smooth, frequently branched at right angles, loosely interwoven, 6–8 µm in diam.; subicular hyphae hyaline, thick-walled, smooth, frequently branched at right angles, 7–11 µm in diam.

***Hymenium***. Cystidioid tubular, hyaline, thin-walled, smooth, with a basal simple septum, occasionally apically obtuse, infrequent, 22–40 × 5–10 μm; basidia slightly barrel-shaped, hyaline, thin-walled, with four to six sterigmata and a basal simple septum, 17–28 × 7–10 μm; basidioles in shape similar to basidia, but slightly smaller.

***Basidiospores***. Fusiform, hyaline, thin-walled, smooth, with one to three guttules, CB+, IKI–, (8.7–)9.4–11.0(–11.7) × (4.7–)4.8–5.9(–6.0) μm, L = 10.29 μm, W = 5.43 μm, Q = 1.90 (n = 50/1).

##### Ecology and distribution.

Growing on angiosperm trunk in the northern subtropical region. So far, known from Anhui Province, China.

## Discussion

In this study, four new species of *Botryobasidium* – *B. fujianensis*, *B. fusisporellum*, *B. macrosporum* and *B. subconiferarum* – are described from east China, based on morphological characters and phylogenetic analyses.

In the phylogeny (Fig. 1), *Botryobasidium fujianensis* is closely related to *B. aureum*, *B. indicum*, *B. coniferarum* and *B. subconiferarum*. However, *B. aureum* differs from *B. fujianensis* by having a white to yellowish hymenial surface when fresh and larger basidiospores (6–9 × 3–4 µm vs. 6–7.9 × 2.3–3.4 µm; Zhou et al. (2024a, 2024b)), whereas *B. coniferarum* differs by the absence of cystidioid and by having wider basidiospores (3.0–3.5 µm vs. 2.3–3.4 µm; Liu et al. (2024)). *Botryobasidium indicum* is reported to possess a velvety, yellow hymenial surface and conidia and chlamydospores, whereas *B. fujianensis* has a floccose to cottony, white to greyish-white hymenial surface and lacks conidia and chlamydospores (Hyde et al. 2019). *Botryobasidium subconiferarum* differs by having basidia with four sterigmata and the presence of conidiophores. Morphologically, *B. candicans* J. Erikss. resembles *B. fujianensis* in having pip-shaped basidiospores, but differs by its narrower basidia (5–7 μm vs. 6–9 μm) and the absence of cystidioid (Bernicchia and Gorjón 2010; Dong et al. 2025). *Botryobasidium conspersum* J. Erikss. resembles *B. fujianensis* in having a greyish-white hymenial surface, but differs by its shorter basidia (12–15 μm vs. 12–20 μm) and longer basidiospores (7–9 μm vs. 6–7.9 μm; Bernicchia and Gorjón (2010); Dong et al. (2025)). *Botryobasidium gossypirubiginosum* Qian Zhou & C.L. Zhao resembles *B. fujianensis* in having a floccose to cottony hymenial surface, but differs by its larger basidia (27.5–28 × 9.5–10 μm vs. 12–20 × 6–9 μm) and basidiospores (14–17.5 × 13–15.5 μm vs. 6–7.9 × 2.3–3.4 μm; Zhou et al. (2024b)).

*Botryobasidium fusisporellum* formed one independent lineage not related to other species in the phylogeny. Morphologically, it resembles *B. chilense* Hol.-Jech. and *B. daweishanense* in having fusiform basidiospores. However, the latter two species have wider basidia (7–8 µm in *B. chilense* and 5–8 µm in *B. daweishanense* vs. 4–7 µm in *B. fusisporellum*) and larger basidiospores (6–7.5 × 3–3.5 µm in *B. chilense* and 6.1–7.3 × 3.3–3.9 µm in *B. daweishanense* vs. 5.3–6.5 × 2.3–3 µm in *B. fusisporellum*). In addition, *B. chilense* lacks cystidioid (Holubová-Jechová 1980; Zhang et al. 2025).

*Botryobasidium subconiferarum* is related to *B. aureum*, *B. fujianensis*, *B. indicum* and *B. coniferarum* in the phylogeny (Fig. 1). However, *B. aureum* differs from *B. subconiferarum* by having a white to yellowish hymenial surface, smaller basidia (12–18 × 6–8 µm vs. 14–22 × 7–10 µm) and basidia with six sterigmata (Zhou et al. 2024a, 2024b); *B. coniferarum* differs by having an arachnoid, whitish to cream hymenial surface and the absence of cystidioid (Liu et al. 2024); and *B. indicum* differs by having a velvety, yellow hymenial surface and possessing chlamydospores reportedly (Hyde et al. 2019). Morphologically, *B. candicans*, *B. curtisii* Hallenb., *B. robustius* Pouzar & Hol.-Jech. and *B. subovalibasidium* L.Jiang Zhou & H.S. Yuan resemble *B. subconiferarum* in having conidiophores. However, *B. candicans* differs by lacking cystidioid and by having narrower basidia (5–7 μm vs. 7–10 μm) with six sterigmata (Bernicchia and Gorjón 2010). *Botryobasidium curtisii* differs by having narrower basidia (6.5–7.5 μm vs. 7–10 μm), longer basidiospores (7.5–9.5 μm vs. 6.2–8.6 μm) and the absence of cystidioid (Hallenberg 1978). *Botryobasidium robustius* differs by lacking cystidioid and by having basidia with six sterigmata (Pouzar and Holubová-Jechová 1967), whereas *B. subovalibasidium* differs by lacking cystidioid and in having larger basidiospores (7–9.8 × 3.7–5 μm vs. 6.2–8.6 × 2.3–3.7 μm; Zhou et al. (2024a)). *Botryobasidium subconiferarum* differs from other species in this genus by its cystidioid and conidiophore; although the effective region of ITS sequence of this species is relatively short (447 bp), it forms a distinct lineage in the phylogenetic tree; therefore, it can be regarded as a new species.

*Botryobasidium macrosporum* is closely related to *B. incanum*, *B. subincanum*, *B. laeve* and *B. vagum* in the phylogeny (Fig. 1). However, *B. incanum* differs from *B. macrosporum* by having basidia with four sterigmata, smaller basidiospores (6.5–8.5 × 3.5–5 µm vs. 9.4–11 × 4.8–5.9 µm) and the absence of cystidioid (Zhou et al. 2024b); *B. laeve* differs by having shorter basidiospores (5–8 µm vs. 9.4–11 µm; Li et al. (2025)); *B. subincanum* differs by having an arachnoid, whitish to ash-grey hymenial surface and smaller basidiospores (7.8–10 × 4–5 µm vs. 9.4–11 × 4.8–5.9 µm; Wang et al. (2025)); and *B. vagum* differs by having a pellicular and greyish hymenial surface and wider basidia (8–12 µm vs. 7–10 µm; Zhou et al. (2024a, 2024b); Li et al. (2025)). Morphologically, *B. candicans*, *B. digitatum* (D.P. Rogers) G. Langer and *B. subovalibasidium* resemble *B. macrosporum* in having similarly shaped basidiospores. However, *B. candicans* differs by having smaller basidia (12–18 × 5–7 µm vs. 17–28 × 7–10 µm) and basidiospores (6–8 × 3–4 µm vs. 9.4–11 × 4.8–5.9 µm) and by lacking cystidioid (Bernicchia and Gorjón 2010). *Botryobasidium digitatum* differs by having a greyish-white hymenial surface and wider basidia (9–12 µm vs. 7–10 µm) with four sterigmata (Hjortstam and Ryvarden 2000). *Botryobasidium subovalibasidium* differs by having smaller basidiospores (7–9.8 × 3.7–5 μm vs. 9.4–11 × 4.8–5.9 μm) and the absence of cystidioid (Zhou et al. 2024a). *Botryobasidium macrosporum* can be easily distinguished from other species in the genus by its cystidioid and larger basidiospores (9.4–11 × 4.8–5.9 µm); and the species forms a distinct lineage in the phylogenetic tree; therefore, it can be regarded as a new species.

Wood-inhabiting fungi play crucial roles in forest ecosystem functioning, but their diversity in China remains insufficiently documented (Dai et al. 2009, 2021; Dai 2010; Wu et al. 2020, 2022a, 2022b; Li et al. 2025). *Botryobasidium* is an important genus of wood-inhabiting fungi that plays a significant role in wood decay. For example, *Botryobasidium botryosum* (Bres.) J. Erikss. possesses diverse enzymes acting on crystalline cellulose (Riley et al. 2014). Furthermore, the *Botryobasidium* may manifest a decay mode that is plesiomorphic for the *Agaricomycetes*, which is important for understanding the origin of wood-inhabiting fungi (Nagy et al. 2016). Due to its important phylogenetic and ecological significance, exploring its diversity is of great importance for future fungal research. This study describes four new species of *Botryobasidium* in east China, thereby expanding the species diversity of the genus and providing a basis for further research on wood-inhabiting fungi in China. In addition, in order to better classify the *Botryobasidium* genus, this study provides a morphological comparison of 20 *Botryobasidium* species with molecular data in China (Table 1), which can provide reference for future research. Species within this genus show considerable morphological diversity and these features have important distinguishing value for taxonomic classification.

**Table 1.** A morphological comparison of 20 *Botryobasidium* species with molecular data in China.

**Species name**	**Hymenial surface**	**Cystidioid**	**Basidia**	**Basidiospores**	**Conidiophores**	
*Botryobasidium acanthosporum*	Smooth, greyish-white to yellowish-white	Present	2 sterigmata; 14.5–20 × 8–10 μm	8–10 × 8–10 μm	Not observed	Zhou et al. (2024a)
*B. bambusinum*	Hypochnoid; rubiginous to slightly reddish-brown	Unknown	Unknown	Unknown	Present	Dong et al. (2025)
*B. coniferarum*	Smooth, arachnoid; whitish to cream	Absent	6 sterigmata; 16–20 × 7–9 µm	7–8 × 3–3.5 µm	Unknown	Liu et al. (2024)
*B. daweishanense*	Araneose; cream	Absent	6 sterigmata; 11.5–18 × 5–8 μm	6.1–7.3 × 3.3–3.9 μm	Unknown	Zhang et al. (2025)
***B. fujianensis***	**Floccose to cottony; white to greyish–white**	**Present**	**4 or 6 sterigmata; 12–20 × 6–9 µm**	**6.0–7.9 × 2.3–3.4 µm**	**Absent**	**Present study**
***B. fusisporellum***	**Floccose to cottony, white to greyish–white**	**Present**	**2 or 4 sterigmata; 11–21 × 4–7 µm**	**5.3–6.5 × 2.3–3 µm**	**Absent**	**Present study**
*B. gossypirubiginosum*	Floccose to cotton; rubiginous	Absent	4 sterigmata; 27.5–28 × 9.5–10 μm	14–17.5 × 13–15.5 μm	Unknown	Zhou et al. (2024b)
*B. incanum*	Hypochnoid, arachnoid; white to incanus	Absent	4 sterigmata; 23–25 × 6–7.5 µm	6.5–8.5 × 3.5–5 µm	Unknown	Zhou et al. (2024b)
*B. indicum*	Velvety; yellow	Unknown	Unknown	Unknown	Present	Hyde et al. (2019)
*B. latihyphum*	Hypochnoid; white to cream	Present	6 sterigmata; 17–25 × 6–8.5 μm	7.9–10.2 × 3.2–4.3 µm	Unknown	Li et al. (2025)
*B. leptocystidiatum*	Arachnoid; greyish white to smoky grey	Present	6 sterigmata; 10.5–15 × 7–8 μm	6.5–7.8 × 2.9–3.7 μm	Not observed	Zhou et al. (2024a)
***B. macrosporum***	**Floccose and granulose; white to cream**	**Present**	**4 or 6 sterigmata; 17–28 × 7–10 μm**	**9.4–11 × 4.8–5.9 μm**	**Absent**	**Present study**
*B. naviculisporum*	smooth, yellowish to pale yellow	Present	4 sterigmata; 14–24 × 6.5–10 µm	12–16 × 6–8 µm	Not observed	Yuan et al. (2026)
***B. subconiferarum***	**Floccose; white to greyish–white**	**Present**	**4 sterigmata; 14–22 × 7–10 μm**	**6.2–8.6 × 2.3–3.7 μm**	**Present**	**Present study**
*B. subincanum*	Smooth, arachnoid; whitish to ash-grey	Absent	6 sterigmata; 18–23 × 8–11 μm	7.8–10 × 4–5 μm	Not observed	Wang et al. (2025)
*B. subovalibasidium*	Smooth; greyish-white to ivory	Absent	4 to 6 sterigmata; 14–18 × 9–10 μm	7–9.8 × 3.7–5 μm	Present	Zhou et al. (2024a)
*B. vagum*	Reticulate to floccose; yellowish to greyish	Absent	6 sterigmata; 20–25 × 8–12 µm	8–12 × 4.5–6 μm	Unknown	Zhou et al. (2024a, 2024b)
*B. xizangense*	Smooth, arachnoid; white	Absent	6 sterigmata; 12–16 × 6–7 µm	7.5–10 × 2.5–3 μm	Not observed	Wang et al. (2025)
*B. yunnanense*	Floccose; buff to slightly yellowish	Absent	6 sterigmata; 25–27 × 4.5–6 µm	11.5–14.5 × 9.5–10.5 µm	Unknown	Zhou et al. (2024b)
*B. zhejiangensis*	Pellicular; white to cream	Absent	6 sterigmata; 15–19 × 5–6 μm	15–19 × 5–6 μm	Unknown	Li et al. (2025)

## Supplementary Material

XML Treatment for Botryobasidium fujianensis

XML Treatment for Botryobasidium fusisporellum

XML Treatment for Botryobasidium subconiferarum

XML Treatment for Botryobasidium macrosporum

## Additional information

### Artificial Intelligence (AI) use

The authors accept full responsibility for the content of the manuscript, including the disclosure of any use of AI.

No AI tools were used in the preparation of this manuscript.

### Author ORCIDs

W. Jing https://orcid.org/0009-0009-8080-9910

W.-Y. Li https://orcid.org/0009-0006-7782-6670

V. Papp https://orcid.org/0000-0001-6994-8156

Q.-X. Guan https://orcid.org/0000-0002-7072-080X

Z.-W. Zheng https://orcid.org/0009-0006-8442-408X

Y.-J. Cui https://orcid.org/0009-0000-8706-6771

F. Wu https://orcid.org/0000-0002-1455-6486

### Data availability

All of the data that support the findings of this study are available in the main text or Supplementary Information.

## Supplementary materials

Supplementary material 1 Maximum Likelihood (ML) phylogenetic tree illustrating the phylogeny of *Botryobasidium*, based on the combined ITS1+5.8S+ITS2 dataset Data type pdf Explanation note Branches are labelled with ML bootstrap values (BS) greater than 75% and Bayesian posterior probabilities greater than 0.90. https://binary.pensoft.net/file/1763703This dataset is made available under the Open Database License (http://opendatacommons.org/licenses/odbl/1.0/). The Open Database License (ODbL) is a license agreement intended to allow users to freely share, modify, and use this Dataset while maintaining this same freedom for others, provided that the original source and author(s) are credited. Wen Jing, Wan-Ying Li, Viktor Papp, Qian-Xin Guan, Zi-Wei Zheng, Yu-Jin Cui, Fang Wu

Supplementary material 2 *Botryobasidium* treebase Data type nxs Explanation note The file named "*Botryobasidium*-treebase" that has been uploaded to TreeBASE. https://binary.pensoft.net/file/1763704This dataset is made available under the Open Database License (http://opendatacommons.org/licenses/odbl/1.0/). The Open Database License (ODbL) is a license agreement intended to allow users to freely share, modify, and use this Dataset while maintaining this same freedom for others, provided that the original source and author(s) are credited. Wen Jing, Wan-Ying Li, Viktor Papp, Qian-Xin Guan, Zi-Wei Zheng, Yu-Jin Cui, Fang Wu
