# Supplementary material for: Morphological characteristics and phylogenetic analyses revealed four new wood-inhabiting species of Botryobasidium (Cantharellales, Basidiomycota) from East China
Source: MycoKeys. 2026 Sep 2;139:321–39. doi: 10.3897/mycokeys.139.206012 (PMC13559518; doi:10.3897/mycokeys.139.206012)
Supplement: Supplementary file 1 [file mycokeys-139-321-s001.pdf]

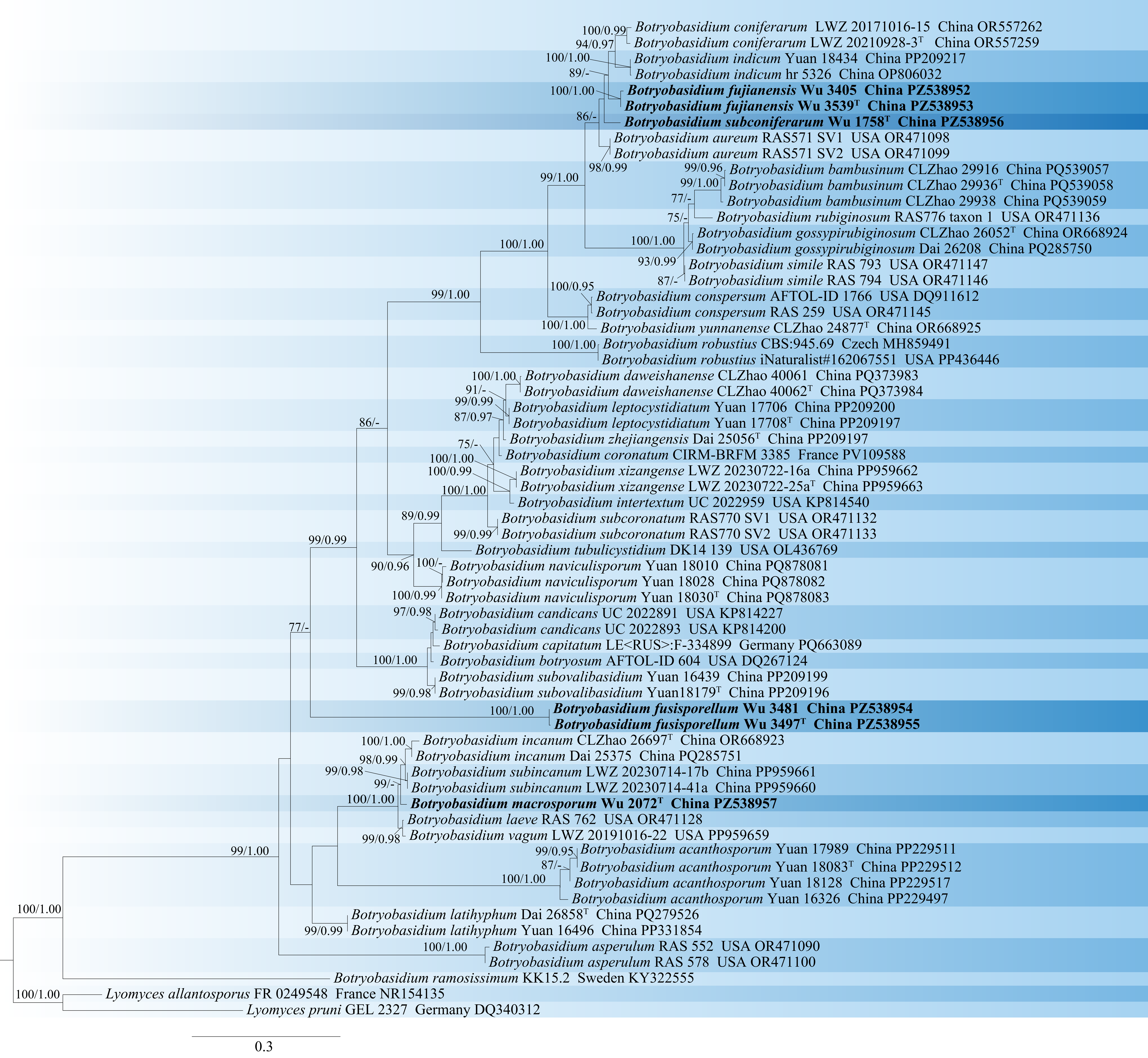
100/0.99
Botryobasidium coniferarum LWZ 20171016-15 China OR557262
94/0.97
Botryobasidium coniferarum LWZ 20210928-3[T] China OR557259
100/1.00
Botryobasidium indicum Yuan 18434 China PP209217
89/-
Botryobasidium indicum hr 5326 China OP806032
100/1.00
Botryobasidium fujianensis Wu 3405 China PZ538952
Botryobasidium fujianensis Wu 3539[T] China PZ538953
86/-
Botryobasidium subconiferarum Wu 1758[T] China PZ538956
Botryobasidium aureum RAS571 SV1 USA OR471098
Botryobasidium aureum RAS571 SV2 USA OR471099
98/0.99
99/0.96
Botryobasidium bambusinum CLZhao 29916 China PQ539057
99/1.00
99/1.00
Botryobasidium bambusinum CLZhao 29936[T] China PQ539058
77/-
Botryobasidium bambusinum CLZhao 29938 China PQ539059
75/-
Botryobasidium rubiginosum RAS776 taxon 1 USA OR471136
Botryobasidium gossypirubiginosum CLZhao 26052[T] China OR668924
100/1.00
100/1.00
Botryobasidium gossypirubiginosum Dai 26208 China PQ285750
93/0.99
Botryobasidium simile RAS 793 USA OR471147
87/-
Botryobasidium simile RAS 794 USA OR471146
99/1.00
100/0.95
Botryobasidium conspersum AFTOL-ID 1766 USA DQ911612
Botryobasidium conspersum RAS 259 USA OR471145
100/1.00
Botryobasidium yunnanense CLZhao 24877[T] China OR668925
100/1.00
Botryobasidium robustius CBS:945.69 Czech MH859491
Botryobasidium robustius iNaturalist#162067551 USA PP436446
100/1.00
Botryobasidium daweishanense CLZhao 40061 China PQ373983
91/-
Botryobasidium daweishanense CLZhao 40062[T] China PQ373984
99/0.99
Botryobasidium leptocystidiatum Yuan 17706 China PP209200
86/-
87/0.97
Botryobasidium leptocystidiatum Yuan 17708[T] China PP209197
Botryobasidium zhejiangensis Dai 25056[T] China PP209197
75/-
Botryobasidium coronatum CIRM-BRFM 3385 France PV109588
100/1.00
100/0.99
Botryobasidium xizangense LWZ 20230722-16a China PP959662
Botryobasidium xizangense LWZ 20230722-25a[T] China PP959663
100/1.00
Botryobasidium intertextum UC 2022959 USA KP814540
89/0.99
Botryobasidium subcoronatum RAS770 SV1 USA OR471132
99/0.99
Botryobasidium subcoronatum RAS770 SV2 USA OR471133
99/0.99
Botryobasidium tubulicystidium DK14 139 USA OL436769
90/0.96
100/-
Botryobasidium naviculisporum Yuan 18010 China PQ878081
Botryobasidium naviculisporum Yuan 18028 China PQ878082
100/0.99
Botryobasidium naviculisporum Yuan 18030[T] China PQ878083
97/0.98
Botryobasidium candicans UC 2022891 USA KP814227
77/-
Botryobasidium candicans UC 2022893 USA KP814200
Botryobasidium capitatum LE<RUS>:F-334899 Germany PQ663089
100/1.00
Botryobasidium botryosum AFTOL-ID 604 USA DQ267124
Botryobasidium subovalibasidium Yuan 16439 China PP209199
99/0.98
Botryobasidium subovalibasidium Yuan18179[T] China PP209196
100/1.00
Botryobasidium fusisporellum Wu 3481 China PZ538954
Botryobasidium fusisporellum Wu 3497[T] China PZ538955
100/1.00
Botryobasidium incanum CLZhao 26697[T] China OR668923
Botryobasidium incanum Dai 25375 China PQ285751
98/0.99
99/0.98
Botryobasidium subincanum LWZ 20230714-17b China PP959661
99/-
Botryobasidium subincanum LWZ 20230714-41a China PP959660
100/1.00
Botryobasidium macrosporum Wu 2072[T] China PZ538957
Botryobasidium laeve RAS 762 USA OR471128
99/0.98
Botryobasidium vagum LWZ 20191016-22 USA PP959659
99/1.00
99/0.95
Botryobasidium acanthosporum Yuan 17989 China PP229511
87/-
Botryobasidium acanthosporum Yuan 18083[T] China PP229512
100/1.00
Botryobasidium acanthosporum Yuan 18128 China PP229517
Botryobasidium acanthosporum Yuan 16326 China PP229497
100/1.00
Botryobasidium latihyphum Dai 26858[T] China PQ279526
99/0.99
Botryobasidium latihyphum Yuan 16496 China PP331854
100/1.00
Botryobasidium asperulum RAS 552 USA OR471090
Botryobasidium asperulum RAS 578 USA OR471100
Botryobasidium ramosissimum KK15.2 Sweden KY322555
100/1.00
Lyomyces allantosporus FR 0249548 France NR154135
Lyomyces pruni GEL 2327 Germany DQ340312
0.3
